# Effects of maternal overnutrition and metabolic challenge in adult life on the histological integrity of the liver and intestinal epithelium in rabbits

**DOI:** 10.3389/fnut.2025.1696494

**Published:** 2025-12-04

**Authors:** Lucía Carolina Cano, Erika Navarrete, Pedro Medina, Juan Pablo Ochoa-Romo, Georgina Díaz, Rodrigo Montúfar-Chaveznava, Rosa María Vigueras-Villaseñor, Ivette Caldelas

**Affiliations:** 1 Instituto de Investigaciones Biomédicas, Universidad Nacional Autónoma de México, Ciudad de México, Mexico; 2 Instituto Nacional de Pediatría, Ciudad de México, Mexico; 3 Escuela Politécnica Superior de Tecnología y Ciencia, Universidad Camilo José Cela, Madrid, Spain; 4 Universidad de Diseño, Innovación y Tecnología, Madrid, Spain

**Keywords:** maternal overnutrition, intestinal epithelium, tissue damage markers, metabolic impairments, high-fat diet, high-carbohydrate diet

## Abstract

**Introduction:**

Maternal overnutrition during critical stages of embryonic development has been implicated as a major determinant of developmental and metabolic disturbances in offspring. Current data concerning how perinatal exposure to high-fat and high-carbohydrate diets, followed by re-exposure in adulthood (metabolic challenge), affects the metabolic regulation and structural integrity of the liver and small intestine, key organs of the gut–liver axis (GLA), are limited.

**Methods:**

Female rabbits were fed either a standard diet (SD) or a high-fat and high-carbohydrate diet (HFCD) before mating and during gestation. The offspring on postnatal day 440 were challenged with an HFCD or SD for 30 days and assigned to the following groups: SD–SD (obtained from does fed SD and challenged with SD), SD–HFCD, HFCD–SD, and HFCD–HFCD. After completion of the metabolic challenge, glucose, total cholesterol, low- and high-density lipoproteins, free fatty acids, triglycerides, gamma-glutamyl transferase, bilirubin, aminotransferases, and the level of lipopolysaccharides (LPS) were measured. Additionally, histological analysis of the liver and duodenum was conducted using several staining methods.

**Results:**

At both metabolic and histopathological levels, rabbits exposed to HFCD during adulthood exhibited more pronounced alterations. In particular, the HFCD–HFCD group showed the greatest degree of impairment. Moreover, morphological changes were notably more severe in the duodenal tissue than in the liver tissue.

**Discussion:**

These results indicate that exposure to an HFCD during critical developmental windows—both gestation and adulthood—induces significant metabolic disturbances and histological damage in the liver and intestinal epithelium. These effects appear to depend on the timing and duration of dietary exposure.

## 1 Introduction

The gut–liver axis (GLA) encompasses a complex bidirectional communication network between the gastrointestinal tract and the liver, resulting from interactions among dietary factors, host genetics, and environmental factors (1). The homeostasis of the GLA is tightly regulated by multiple components, including intestinal microbiota composition, gut epithelial barrier integrity, antigen translocation, mucosal innate immune responses, and oxidative stress, as well as non-digested or malabsorbed nutrients (2). Increasing evidence indicates that disturbances to the GLA, such as dysbiosis and increased intestinal mucosa permeability (commonly referred to as “leaky gut”), trigger hepatic inflammatory pathways primarily via Toll-like receptor (TLR) signaling in hepatocytes. This activation exacerbates oxidative stress and inflammatory responses, thereby contributing to the onset and progression of several hepatic pathologies (3).

The dynamic interplay between the liver and the intestine plays an essential role in both health and disease development. Dietary patterns critically influence the integrity and function of the GLA. Emerging evidence indicates that high-fat diets (HFDs) are associated with an aberrant microbiome, whereas lifestyle habits such as regular physical activity and a healthy diet, including the Mediterranean diet, promote beneficial modulation of both the diversity and abundance of the gut microbiota (4). The GLA is characterized by bidirectional communication, in which nutrients and microbiota-derived metabolites are conveyed to the liver predominantly via the portal circulation. Moreover, the liver synthesizes and secretes bile acids to facilitate lipid emulsification, which in turn promotes their intestinal absorption and subsequent enterohepatic recirculation. This reciprocal relationship is critical in modulating metabolic processes and plays a significant role in the onset and progression of liver diseases (5).

Experimental studies have demonstrated that high-fat and high-carbohydrate diets (HFCDs) negatively affect metabolic homeostasis, promoting obesity and alterations in serum biochemical parameters (6, 7). Moreover, these diets disrupt the diversity and composition of the microbiota and compromise gut barrier integrity, leading to increased intestinal permeability (leaky gut), facilitating intrahepatic lipid accumulation and ultimately contributing to the pathogenesis of hepatic inflammation and fibrosis (8, 9). Similarly, murine models of maternal obesity induced by a Western diet have shown an elevated risk of liver disease in offspring persisting into adulthood (10). Furthermore, rodents fed a cafeteria-type diet exhibit adaptive intestinal modifications, including an increased absorptive surface area of the small intestine, a greater proportion of goblet cells, and an elevated inflammatory index within the duodenum (11). Additionally, the pronounced hepatic inflammation reported in mice exposed to an HFD has been mechanistically linked to disruptions in the GLA, specifically through compromised integrity of the small intestinal mucosa (12).

The liver is a vital multifunctional organ involved in macronutrient metabolism, regulation of blood volume, cholesterol and lipid homeostasis, energy production, and xenobiotic metabolism, among numerous other physiological functions (13). In parallel, the intestinal epithelium acts as a dynamic interface between the external environment and the internal milieu. The integrity of the intestinal barrier is maintained by a coordinated network of specialized cells, including enterocytes, goblet cells, and immune cells, which are tightly interconnected by junctional complexes and covered by a mucus layer that serves as a selective barrier and works in a coordinated manner to maintain intestinal homeostasis (2, 14–16).

Currently, acute and chronic liver diseases constitute a growing global health concern, responsible for approximately 2 million deaths annually and accounting for 4% of total global mortality (17, 18). Similarly, inflammatory bowel disease (IBD) has emerged as a significant global burden, with an increasing incidence and a prevalence exceeding 0.3% in Western countries (19). Epidemiological trends underscore the critical need to deepen our understanding of disruptions in the GLA, particularly in the context of metabolic and inflammatory disorders.

During early developmental stages, nutrition plays a central role in shaping long-term metabolic outcomes through a process known as metabolic programming (20). The Developmental Origins of Health and Disease (DOHaD) hypothesis, initially proposed by Barker, posits that during critical windows of development, organisms are particularly susceptible to environmental insults, which may predispose them to an increased risk of metabolic diseases later in life (21, 22). A growing body of evidence suggests that intrauterine environmental conditions significantly influence offspring susceptibility to chronic non-communicable diseases. In particular, maternal overnutrition due to chronic intake of an HFCD has been linked to a high risk of developing hypertension, cardiovascular disease, type 2 diabetes, obesity, neurocognitive dysfunction, non-alcoholic fatty liver disease, and metabolic syndrome in adult offspring (22). Furthermore, excessive nutrient intake during gestation has been associated with the dysregulation of hepatic molecular pathways that promote hepatic steatosis, and the upregulation of biomarkers related to metabolic dysfunction and liver disease (23, 24). These findings strongly indicate the long-lasting impact of maternal diet on offspring health, particularly through modulation of the GLA and its association with metabolic processes.

Adults raised in obesogenic environments often adopt and perpetuate the eating habits modeled by their parents, reflecting an intergenerational transmission of nutritional behaviors (25). In support of this, experimental studies in rodents have demonstrated that offspring born to dams fed HFCD during gestation exhibit a postweaning preference for similarly unbalanced diets (26). Such dietary preferences can persist into adulthood (27), suggesting a long-term impact of early nutritional exposure on feeding behavior. Based on these findings, our research group has established a model of maternal overnutrition to investigate the effects of unbalanced dietary intake during pregnancy on metabolic programming and long-term health outcomes in rabbits (28–30). The aim of this study was to evaluate whether chronic maternal intake of an HFCD throughout gestation, combined with subsequent dietary exposure in adult offspring, alters the metabolic profile and induces morphological changes in key organs associated with metabolic regulation, the liver and duodenum. We hypothesized that exposure to a high-fat and high-carbohydrate diet (HFCD) during gestation would induce alterations in liver and gut morphology and metabolic disturbances in the offspring. Furthermore, we proposed that liver and gut morphology, along with overall metabolic status, would also be influenced by the type of diet—standard diet (SD) or HFCD—ingested during adulthood.

## 2 Materials and methods

All the experimental procedures were conducted in accordance with the guidelines of the National Institutes of Health, the Guide for the Care and Use of Laboratory Animals (NIH Pub. No. 86–23, revised in 1996), and the research guidelines of the Biomedical Research Institute, National Autonomous University of Mexico (UNAM). The Animal Care and Use Committee of the Biomedical Research Institute, UNAM, approved the protocol before the study was conducted (ID: 198).

This study was conducted using domestic Chinchilla rabbits (*Oryctolagus cuniculus*) maintained under controlled environmental conditions as previously described in our earlier studies (29, 30). The rabbits were housed individually in stainless-steel cages (120 × 60 × 45 cm) under a 16:8 h light–dark cycle (lights on at 09:00 h) at a constant room temperature (20 ± 2 °C), with the relative humidity maintained between 40 and 60%.

### 2.1 Diet and experimental design

The animals in this study were fed a standard diet (SD; Conejo Ganador, Malta Cleyton, Mexico) formulated to provide 2,542.6 kcal/kg, with 3.8% kcal of fat and 47.8% kcal of carbohydrates, or a high-fat and high-carbohydrate energy-dense diet (HFCD). The HFCD was prepared using the SD, which was supplemented with 0.1% cholesterol (Sigma, United States), 4% soybean oil (Sigma, USA), and 15% sucrose (Great Value, Mexico), providing 2,609.2 kcal/kg and containing 5.6% kcal fat and 52.6% kcal carbohydrates (47 and 10% more energy from fats and carbohydrates, respectively) than SD (29, 30).

**Figure 1:**
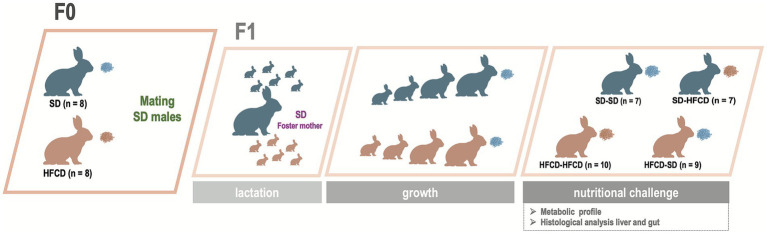
Experimental design and dietary interventions in does and offspring. Female rabbits (F0) were fed either a standard diet (SD) or a high-fat and high-carbohydrate diet (HFCD) and subsequently mated with SD-fed male rabbits. The timeline illustrates the follow-up and analysis of offspring (F1). Offspring obtained from SD- and HFCD-fed does were nursed by SD-foster mothers and subsequently weaned onto an SD. At postnatal day 440, the offspring were divided into four groups according to maternal and adult dietary exposure: SD/SD, SD/HFCD, HFCD/SD, and HFCD/HFCD. The nutritional challenge lasted 30 days, after which the animals were euthanized, and blood, liver, and duodenum samples were collected for metabolic and histological assessment.

Offspring were obtained from 10-week-old nulliparous female rabbits (*n* = 16), which were randomly assigned to one of the following groups: the F0-SD group (*n* = 8), which was fed a standard rabbit diet, or the F0-HFCD group (*n* = 8), which was fed an HFCD. The supplemented diet was provided for 8 weeks before mating (Figure 1). Food and water were available *ad libitum*. At 140 days of age, the female rabbits were mated with 170-day-old male rabbits maintained on an SD diet throughout their lifetime. During pregnancy, does in the SD group continued to receive SD exclusively, whereas the HFCD-fed does were alternately provided with an HFCD or SD to avoid pregnancy complications and prevent miscarriages, as previously described (29–31).

Four days before parturition, artificial burrows were installed in each cage to facilitate nest building, and sterile hay was provided. The burrows (28 × 29.5 × 30 cm high) were constructed from opaque polyvinyl chloride, with a 14 cm diameter entrance to mimic natural nesting conditions. At birth, the rabbit pups were weighed and marked for individual identification. Although both groups exhibited a comparable female-to-male offspring ratio at birth (SD: 42.3/57.69%; HFCD: 42.16/57.8%), only male offspring were included in the present study. Litter size was standardized to six pups per dam.

To ensure uniformity in early postnatal development, offspring from all the experimental groups were nursed exclusively by foster does fed SD until postnatal day (PND) 31. Following weaning at PND 36, all F1 rabbits were provided *ad libitum* access to SD and water.

To assess whether maternal dietary exposure before and during gestation modulates offspring responses to a postnatal nutritional challenge, F1 rabbits from both maternal groups were challenged with an HFCD in adulthood. At 440 days of age, the animals were randomly assigned to one of four groups based on maternal and postnatal challenge diets: SD/SD (*n* = 7), SD/HFCD (*n* = 7), HFCD/SD (*n* = 9), or HFCD/HFCD (*n* = 10). The nutritional challenge consisted of either continued SD or the introduction of an HFCD for 30 days (Figure 1).

After the nutritional challenge, all the animals were weighed and subsequently euthanized via inhalation of 3 mL of sevoflurane (Abbott, Mexico), followed by intravenous administration of an overdose of pentobarbital (30 mg/kg, Pisabental^®^, Pisa Agropecuaria, Mexico) into the marginal vein of the left ear. The liver and duodenum were carefully dissected. Liver samples were immediately weighed, frozen in liquid nitrogen, and stored at −80 °C until further analysis. Duodenal tissue was gently rinsed with sterile isotonic saline solution (0.9% NaCl; Laboratorios Pisa, Guadalajara, Mexico), placed in histologic cassettes, and preserved in formalin (4% formaldehyde; JT Baker, Mexico) until histological tissue processing.

### 2.2 Metabolic assessment and damage markers

To assess metabolic profiles, plasma lipoprotein concentrations, and biomarkers of tissue damage following a metabolic challenge, blood samples were collected after 15 h of fasting (food was withdrawn while water remained accessible). The rabbits were immobilized with a snuggle restraint, and approximately 3 mL of blood was drawn from the central auricular artery using a sterile 24-G catheter (Terumo, Japan). The blood was collected in plastic serum tubes coated with silica (BD Vacutainer, Canada) and centrifuged (3,000 rpm, 15 min, room temperature). The serum was aliquoted and stored at −70 °C until biochemical analysis. The following parameters were quantified using spectrophotometric methods (29, 30): glucose (GLU), total cholesterol (CHOL), low-density lipoprotein cholesterol (LDL-C), high-density lipoprotein cholesterol (HDL-C), free fatty acids (FFA), glycerol (GLY), triglycerides (TG), gamma-glutamyl transferase (GGT), total bilirubin (TB), conjugated bilirubin (CB), aspartate aminotransferase (AST), and alanine aminotransferase (ALT). All analyses were performed using commercial enzymatic colorimetric assay kits (Randox Laboratories Ltd., United Kingdom; Biosino Biotechnology & Science Inc.), according to the manufacturers’ instructions.

To quantify the serum levels of lipopolysaccharides (LPS), an enzyme-linked immunosorbent assay (ELISA) was performed using the LPS ELISA kit (orb440611, Biorbyt NC, United States), which has a detection range of 3.12–200 ng/mL. The assay was performed according to the manufacturer’s instructions. Briefly, 550 μL of serum was mixed with 50 μL of Reagent A and 100 μL of Reagent B. Following incubation, 90 μL of substrate solution was added, and the enzymatic reaction was terminated with 50 μL of stop solution. The absorbance was measured at 450 nm using a Multiskan Go microplate spectrophotometer (Thermo Fisher Scientific, Vantaa, Finland).

### 2.3 Liver histology

To evaluate hepatic damage at the histological level, livers obtained from dissection were immediately frozen in liquid nitrogen and stored at −80 °C until further analysis. Liver tissue was processed as follows: tissue blocks of approximately 2.5 × 3 cm were fixed with 4% formalin for 48 h (freezing the tissue and fixation with formalin ensure lipid preservation). The samples were subsequently cryoprotected by immersion in increasing concentrations of sucrose solutions (10, 20, and 30%) for 48 h at each concentration. Liver sections were obtained using a cryostat (Microm HM520, International GmbH, Germany), and serial 7-μm-thick samples were collected from the main hepatic region at −27 °C. The sections were mounted on microscope slides (Corning, NC, United States, 2947 N-25×75) and air-dried for 24 h before staining.

To assess hepatic structure and pathological alterations, hematoxylin and eosin (HE) staining was performed to identify hepatic architecture, including hepatocytes, sinusoidal spaces, and hepatic nuclei, as well as the presence of macrovesicles. To determine the presence of collagen fibers, Masson’s trichrome (MT) staining was used to assess collagen fiber deposition, a marker of fibrosis. To identify and assess lipid accumulation, lipid droplets were stained with Oil Red O (ORO).

In liver sections, hepatic acinar zones were delineated and analyzed to assess the presence of macrovesicular and microvesicular steatosis and collagen deposition. Semiquantitative scoring was used for all features: absence (0), low (1), mild (2), and high (3). Individual scores obtained for each histological feature were summed across the acinar zones within each group, and the results are expressed as percentages.

### 2.4 Duodenal histology

To evaluate morphological alterations in the intestinal epithelium, a 1-cm sample of the duodenum was fixed in 4% paraformaldehyde (Sigma-Aldrich, Mexico) and washed with PBS (3 × 30 min). The samples were processed using a fully automated tissue processor (Leica TP1020, Leica Biosystems, Nussloch, Germany), with sequential dehydration in a graded ethanol series up to 100%.

Once the tissue was fully dehydrated, the samples were embedded in paraffin blocks, and serial 5-μm-thick sections of the duodenum were obtained using a rotary microtome (Leica RM 2155, Leica Biosystems, Nussloch, Germany). The tissue sections were mounted on microscope slides (Corning 2,947 N-25×75) and dried at room temperature for 24 h before further histological staining.

Hematoxylin–eosin staining was used to assess the histological structure and quantify morphological parameters, including villus length and width, goblet cell count per villus, muscle thickness, and the number and area of Brunner glands. MT staining was used to detect and evaluate collagen fibers. To identify goblet cells, Periodic Acid–Schiff (PAS) staining was performed.

For both tissues (duodenum and liver) and all staining analyses (HE, ORO, MT, PAS), morphometric measurements were conducted using the ImagePro 6 software (Media Cybernetics, United States) coupled with a brightfield microscope (Olympus BX51, Life Science Solutions, USA) equipped with a digital single-lens reflex camera (Canon^®^ EOS Rebel T5i, United States). All measurements were performed under a single-blind protocol, in which a single evaluator performed histological assessments without being aware of the identity of each animal or the group to which it belonged. Another researcher was responsible for data organization and categorization.

The sample sizes for each group for all analyses were as follows: SD/SD (*n* = 7), SD/HFCD (*n* = 7), HFCD/SD (*n* = 9), and HFCD/HFCD (*n* = 10). All histological staining procedures were performed following established protocols (32, 33). Detailed information regarding the measurement parameters (amplification, number of sections, number of measurements, and specifications for each measurement) is summarized in Table 1.

**Table 1:** Summary of histological measurements in the gut and liver of rabbits.

Stain	Measurement	Amplification	Number of measurements	Description of measurements
Duodenum				
HE	Villi length	4x	9 different villi per animal, 3 times each villi	From the tip of the villus to the base, where it connects to the intestinal crypt. Measurement area: 2,230 × 1,464 μm.
	Villi width	4x	9 different villi per animal, 3 times each villi	At the midpoint of a villus, extending from one side to the other across the villus. Measurement area: 2,230 × 1,464 μm.
	Muscle thickness	10x	9 different measurements of muscle layers per animal, 3 times each section	At the midpoint of the muscle layer. Measurement area: 894 × 585 μm.
	Brunner gland area	10x	3 measurements of each Brunner’s gland	The perimeter of each gland was traced to obtain the area of each gland. Measurement area: 894 × 585 μm.
	Brunner gland quantification	10x	Quantification of Brunner glands in the specified area, 3 times each measurement	Quantification of glands found in the specified area. Measurement area: 894 × 585 μm.
	Crypt depth	10x	9 measurements of crypt depth per animal, 3 times each crypt	By the depth of the invagination between adjacent villi. Measurement area: 894 × 585 μm.
PAS	Goblet cell quantification	10x	Quantification of the goblet cells in the specified area, 3 times each measurement	Quantification of all blue-stained cells in each section. Measurement area: 894 × 585 μm.
MT	Collagen	10x	3 measurements per each duodenal zone submucose mucose muscularis propia serose	Crosses were used to determine mild (x), moderate (xx), and abundant (xxx) collagen. Each cross score depends on the area where it is found: 0: absence of collagen fibers 1: presence in submucosa and mucosa 2: presence in muscularis mucosa and muscularis propria 3: presence in all layers, including muscularis propria and serosa Average score per group was obtained (77, 78) Measurement area: 894 × 585 μm.
Liver				
HE	Macrovesicles	20x	3 measurements per each acinus zone (1,2,3)	The tissue was stained with HE and quantified the holes formed in each section.
ORO	Microvesicles	20x	3 measurements per each acinus zone (1,2,3)	Red-stained lipid droplets were quantified in each section (six histological sections).
MT	Collagen	4x	3 measurements per each acinus zone (1,2,3)	The following was applied: score: absence (0), low (1), mild (2), high (3). the average score per group was obtained.

HE, hematoxylin–eosin, MT, Masson’s trichrome stain, ORO, Oil Red O staining, PAS, Periodic Acid–Schiff.

### 2.5 Data analysis

Serum metabolite concentrations and circulating LPS levels were analyzed using one-way analysis of variance (ANOVA) for independent measures to evaluate potential differences attributable to dietary exposure, followed by Fisher’s least significant difference (LSD) *post-hoc* test, with a significance threshold set at *p* < 0.05. Markers of hepatic injury were analyzed via the non-parametric Kruskal–Wallis test, followed by Dunn’s *post-hoc* comparisons. We performed a Spearman’s correlation analysis to assess the relationship between the metabolic profile and circulating markers of liver damage.

Histomorphometric parameters of the duodenum (villi length and width, muscle thickness, crypt depth, number of goblet cells, Brunner’s glands, and lipid droplets) were analyzed via one-way ANOVA, followed by Fisher’s *post-hoc* test. The Kruskal–Wallis test, followed by Dunn’s *post-hoc* test with false discovery rate (FDR) correction, was employed to analyze semiquantitative scores for hepatic macro- and microvesicular steatosis, collagen deposition, and intestinal collagen content.

The statistical analyses were conducted using StatView (SAS Institute, Inc.) and the Software R base (version 4.5.0). Spearman’s association analysis was performed in R, and the “Psych” package was used to determine statistical significance. The visualization was performed with the “corrplot2” package. The graphical representations were generated in SigmaPlot software (Grafiti, CA).

## 3 Results

### 3.1 Metabolic assessment and damage markers

**Figure 2:**
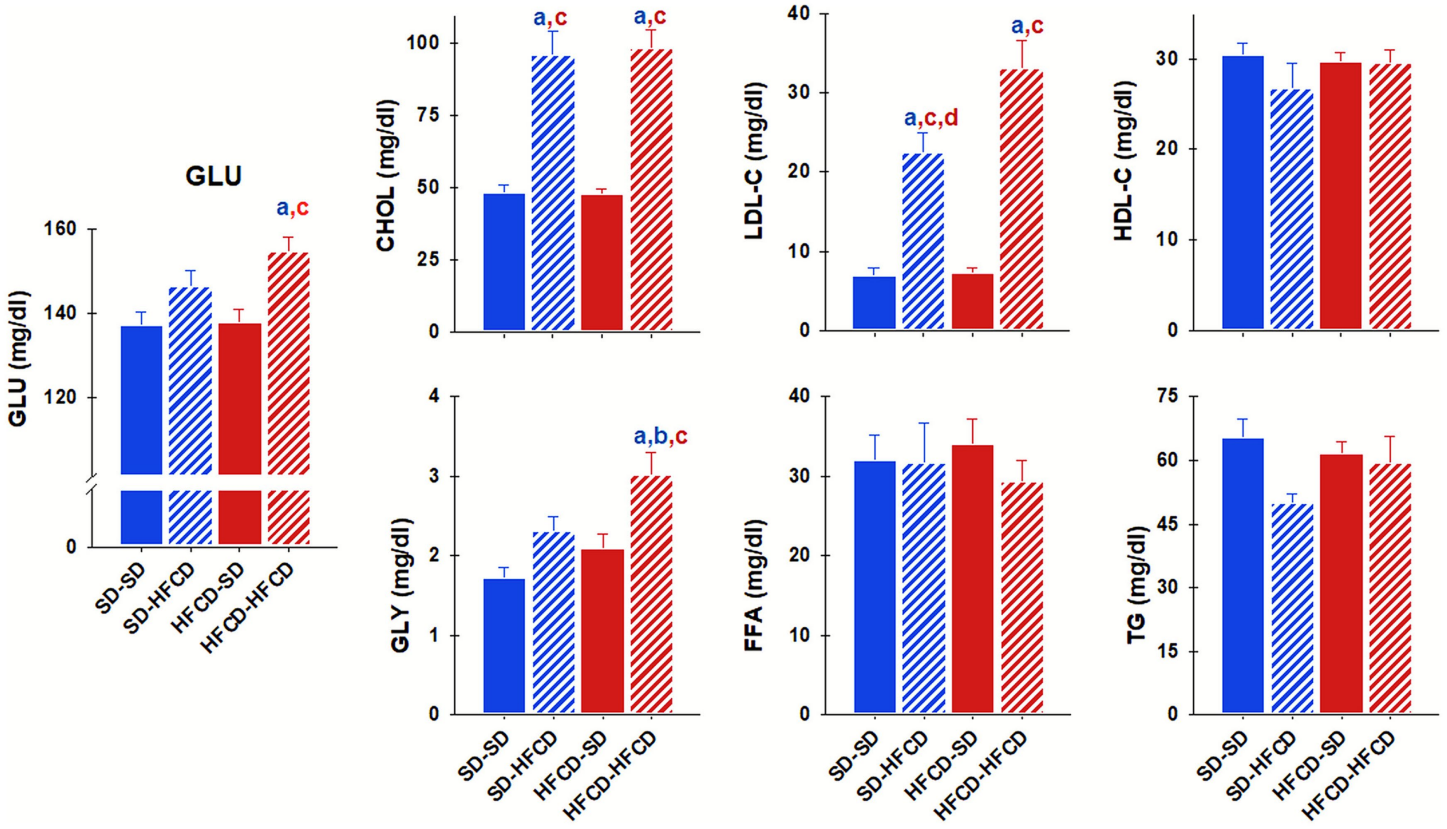
Metabolic profile. Plasma levels of glucose (GLU), total cholesterol (CHOL), low-density lipoprotein cholesterol (LDL-C), high-density lipoprotein cholesterol (HDL-C), glycerol (GLY), free fatty acids (FFAs), and triglycerides (TGs) were measured in adult rabbits obtained from mothers fed a standard diet (SD; blue bars) or a high-fat and high-carbohydrate diet (HFCD; red bars). In adulthood, the offspring were metabolically challenged with either the SD or the HFCD (striped blue and red bars). Experimental groups: SD/SD (*n* = 7), SD/HFCD (*n* = 7), HFCD/SD (*n* = 9), and HFCD/HFCD (*n* = 10). Mean ± SEM. *a* indicates *p* < 0.05 vs. SD/SD; *b* vs. SD/HFCD; *c* vs. HFCD/SD.

After the metabolic challenge, important effects at the metabolic level were observed between the groups. The serum GLU levels were significantly different between the groups [*F*_(3, 71)_ = 6.8; *p = <*0.0001], and the HFCD–HFCD group showed significantly elevated GLU concentrations (*p* < 0.0001), approximately 12.62% higher than those in the SD–SD group. In terms of lipid metabolism, CHOL levels also differed significantly between groups [*F*_(3, 72)_ = 33.4; *p* < 0.0001], and the HFCD–HFCD and SD–HFCD groups exhibited marked increases of approximately 103 and 98%, respectively, compared with the SD–SD group (Figure 2). Similarly, LDL-C plasma levels were significantly affected by dietary conditions [*F*_(3, 70)_ = 28.7; *p* < 0.0001], with increases of 370 and 220% observed in the HFCD–HFCD and SD–HFCD groups, respectively, compared with those in the SD–SD group. The GLY levels significantly differed between the experimental groups [*F*_(3, 27)_ = 6.7; *p* = 0.001], with a 74% increase in the HFCD–HFCD group compared with the SD–SD group (Figure 2). In contrast, the serum levels of FFA, TG, and HDL-C did not significantly differ among the groups (Figure 2).

**Figure 3:**
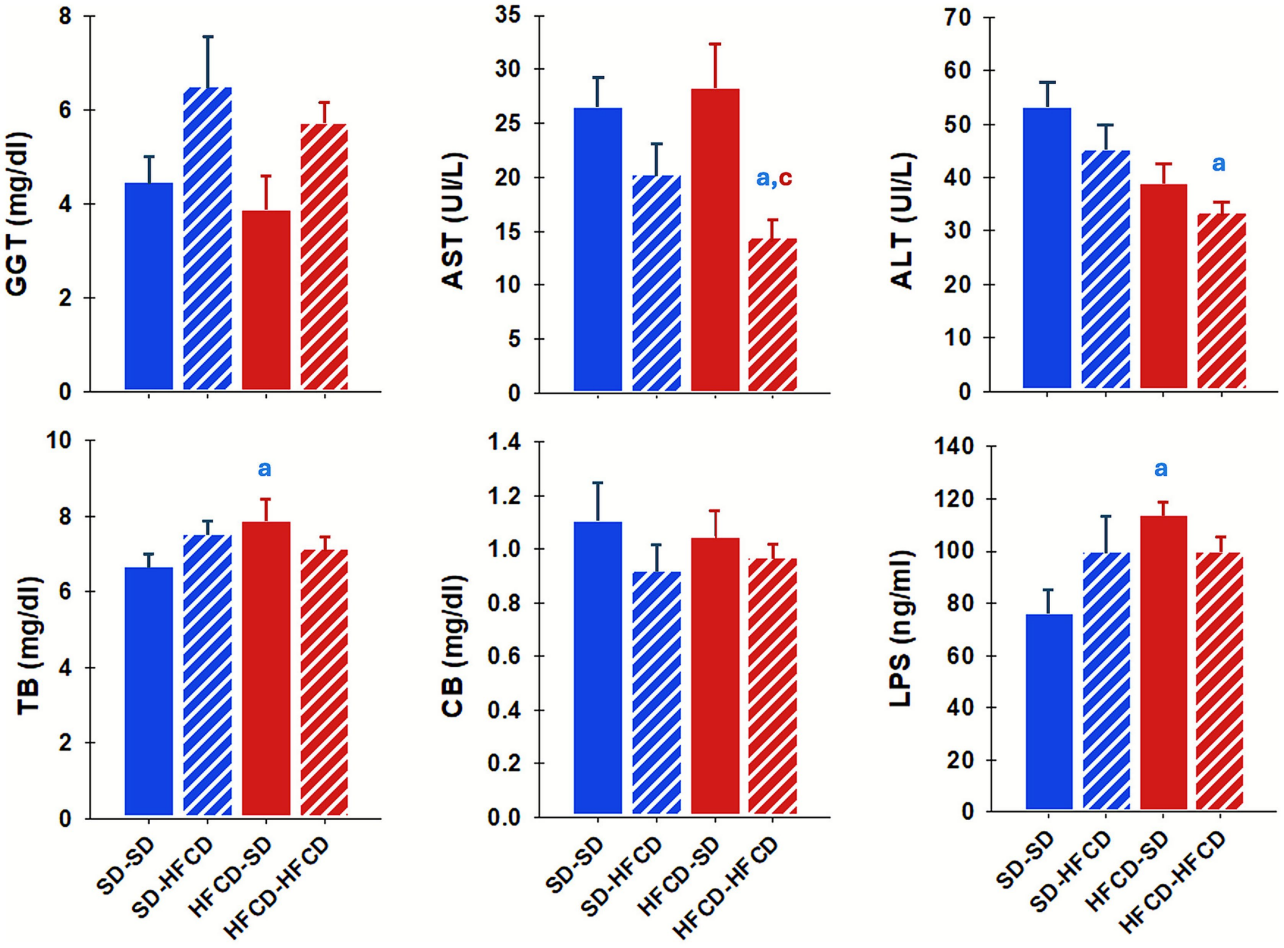
Markers of tissue damage. Plasma concentrations of gamma-glutamyl transferase (GGT), aspartate aminotransferase (AST), alanine aminotransferase (ALT), total bilirubin (TB), conjugated bilirubin (CB), and lipopolysaccharides (LPS) in adult rabbits obtained from mothers fed a standard diet (SD; blue bars) or a high-fat and high-carbohydrate diet (HFCD; red bars). In adulthood, the offspring were metabolically challenged with either the SD or the HFCD (striped blue and red bars). Experimental groups: SD/SD (*n* = 7), SD/HFCD (*n* = 7), HFCD/SD (*n* = 9), and HFCD/HFCD (*n* = 10). Mean ± SEM. *a* indicates *p* < 0.05 *vs*. SD/SD; *b vs*. SD/HFCD; *c vs*. HFCD/SD.

Significant alterations in the serum markers of hepatic injury, TB (*H* = 9.9; *p* = 0.02), AST (*H* = 17.6; *p* < 0.001), and ALT (*H* = 13.1; *p* = 0.005), were observed in response to maternal overnutrition and/or postnatal metabolic challenge (Figure 3). Notably, the HFCD–SD group presented significantly greater TB levels (approximately 17.5%) than those in the SD–SD group. Conversely, the HFCD–HFCD group showed significantly lower AST (45.6%) and ALT (37%) levels than those in the SD–SD group. No significant differences attributable to maternal nutrition or dietary challenge were observed for GGT or CB (Figure 3).

In addition, LPS levels were significantly different between the experimental groups [*F*_(3, 22)_ = 3.9; *p* = 0.02]. Compared with the SD–SD group, the HFCD–SD group showed a significant increase in LPS of approximately 48.8% (Figure 3).

### 3.2 Correlation analysis of metabolites and liver damage markers

**Figure 4:**
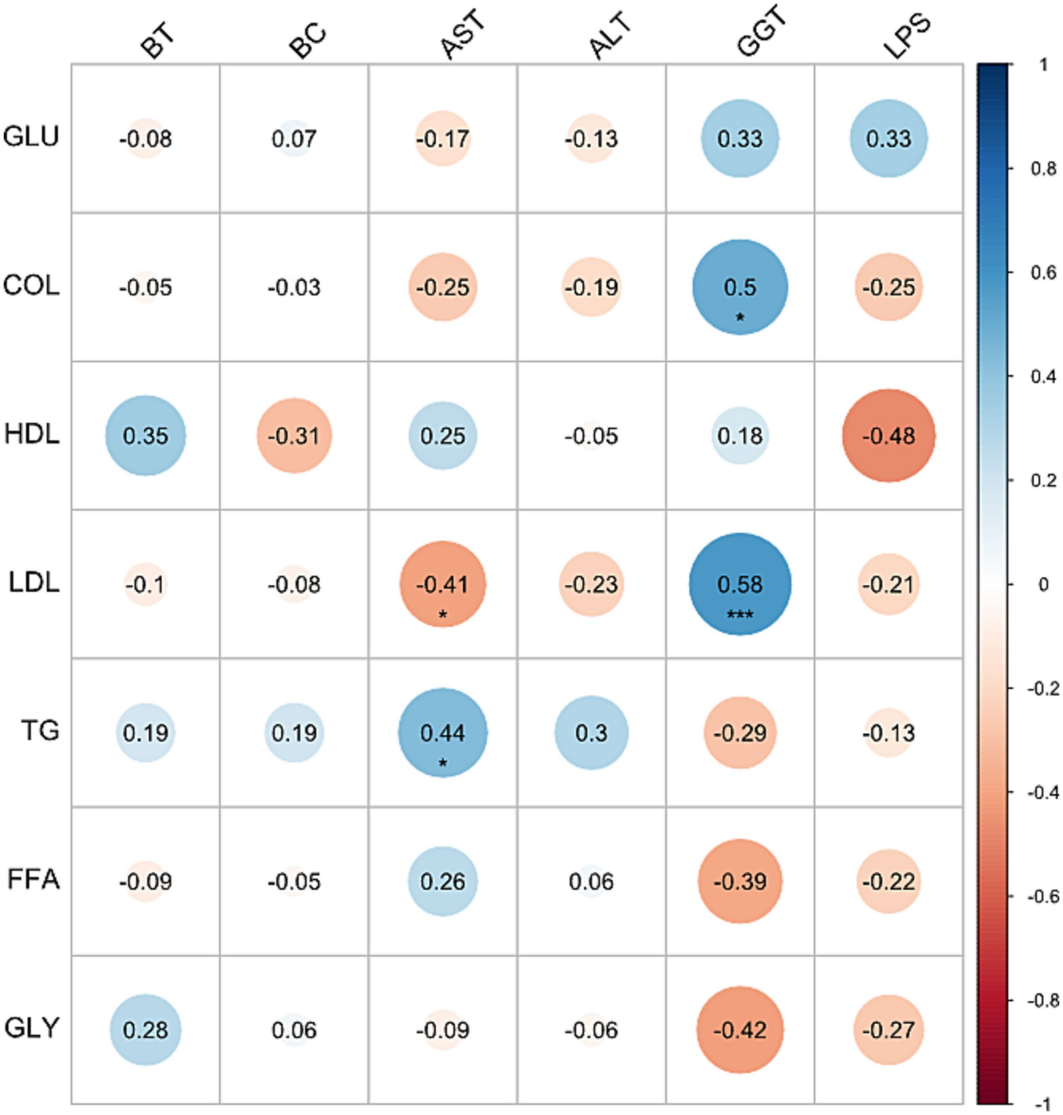
Spearman’s correlation between metabolites and liver damage markers. Association between plasma levels of metabolites—glucose (GLU), total cholesterol (CHOL), low-density lipoprotein (LDL), high-density lipoprotein (HDL), glycerol (GLY), free fatty acids (FFAs), and triglycerides (TGs)—and liver damage markers: gamma-glutamyl transferase (GGT), aspartate aminotransferase (AST), alanine aminotransferase (ALT), total bilirubin (TB), conjugated bilirubin (CB), and lipopolysaccharides (LPS) in adult rabbits obtained from mothers fed a standard diet (SD) or high-fat and high-carbohydrate diet (HFCD). In adulthood, the offspring were metabolically challenged with either the SD or the HFCD. Experimental groups: SD/SD (*n* = 7), SD/HFCD (*n* = 7), HFCD/SD (*n* = 9), and HFCD/HFCD (*n* = 10). *p < 0.05, ***p < 0.005.

Spearman’s coefficient (Figure 4) revealed a significant negative association of AST with LDL (*r* = −0.41; *p* < 0.05) and a positive association with TG (*r* = 0.44; *p* < 0.05). Furthermore, GGT was positively associated with both CHOL (*r* = 0.5; *p* < 0.001) and LDL (*r* = 0.58; *p* < 0.05).

### 3.3 Liver and duodenum histology

**Figure 5:**
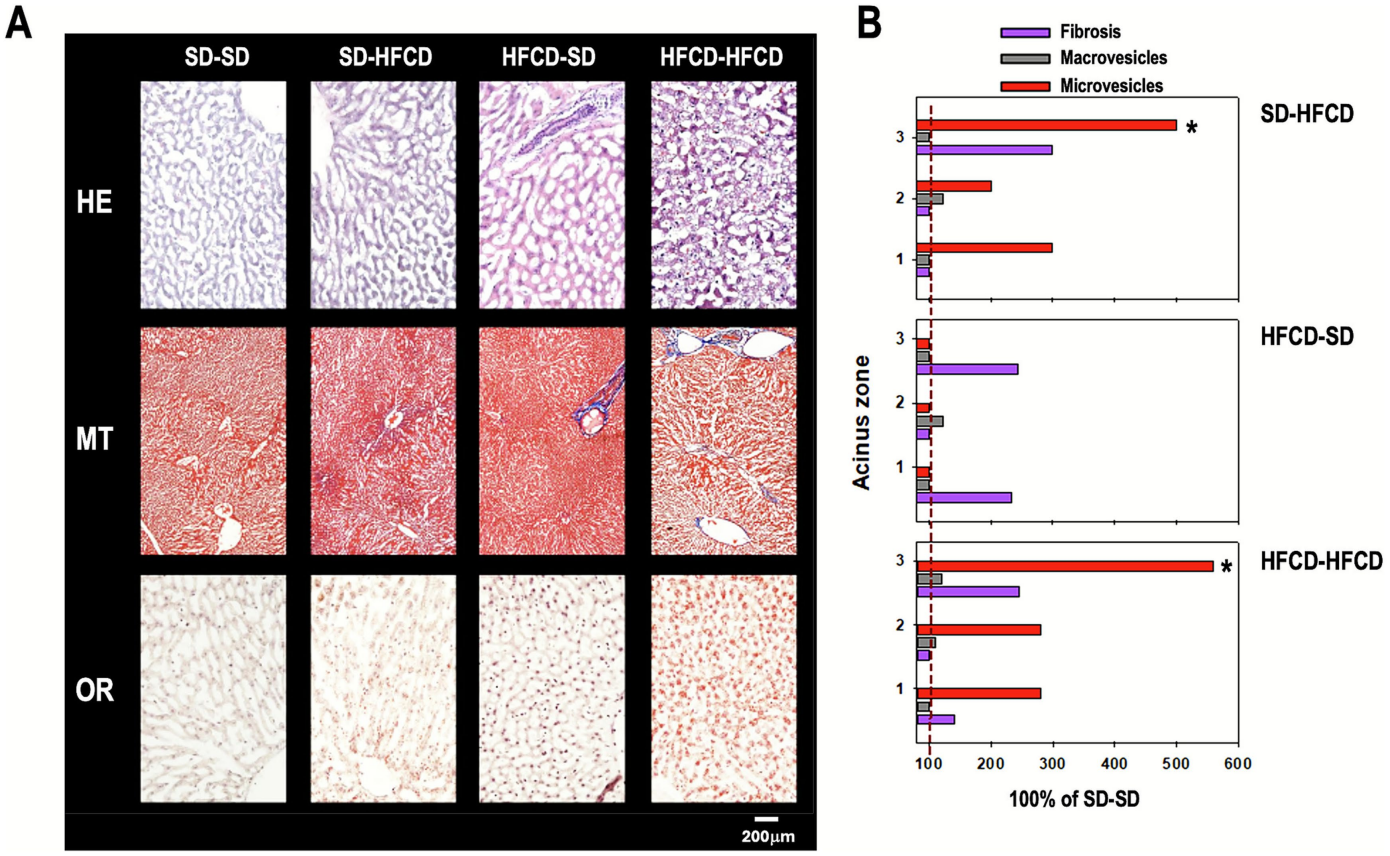
Liver histology. **(A)** Photomicrograph of representative histological images of the liver. **(B)** Quantification of macro- and microvesicles, and collagen fibers in the three hepatic acinus zones in offspring obtained from mothers fed a standard diet (SD) or a high-fat and high-carbohydrate diet (HFCD). In adulthood, the offspring were metabolically challenged with either the SD or the HFCD. The horizontal bars indicate the fold change between the values obtained from the SD group. Sections were stained with hematoxylin–eosin (HE, 20×), Masson’s trichrome (MT, 4×), and Oil Red O (ORO, 20×) in rabbit liver tissue. *p < 0.05,

At the histological level, in the hepatic acinus, significant alterations in the presence of microvesicles were identified in Zone 3 (Zone 1: *H* = 5.8; *p =* 0.1; Zone 2: *H* = 13.2; *p =* 0.004; Zone 3: *H* = 16.6, *p* < 0.001). Compared with the SD–SD group, both the SD–HFCD and HFCD–HFCD groups showed statistically significant increases in microvesicle accumulation of approximately 500 and 570%, respectively (Figure 5).

With respect to the presence of macrovesicles in the liver (Figure 5), no statistically significant differences were observed among the experimental groups in any of the zones (Zone 1: *H* = 5.5, *p* = 0.1; Zone 2: *H* = 3.2, *p* = 0.4; Zone 3: *H* = 4.7, *p* = 0.2). Similarly, MT staining revealed no significant differences in collagen fiber deposition across the three hepatic zones (Zone 1: *H* = 7.6, *p* = 0.06; Zone 2: *H* = 0, *p* = 1.000; Zone 3: H = 5.2, *p* = 0.2), indicating that neither maternal overnutrition nor nutritional challenge induced detectable fibrotic alterations in the liver (Figure 5).

**Figure 6:**
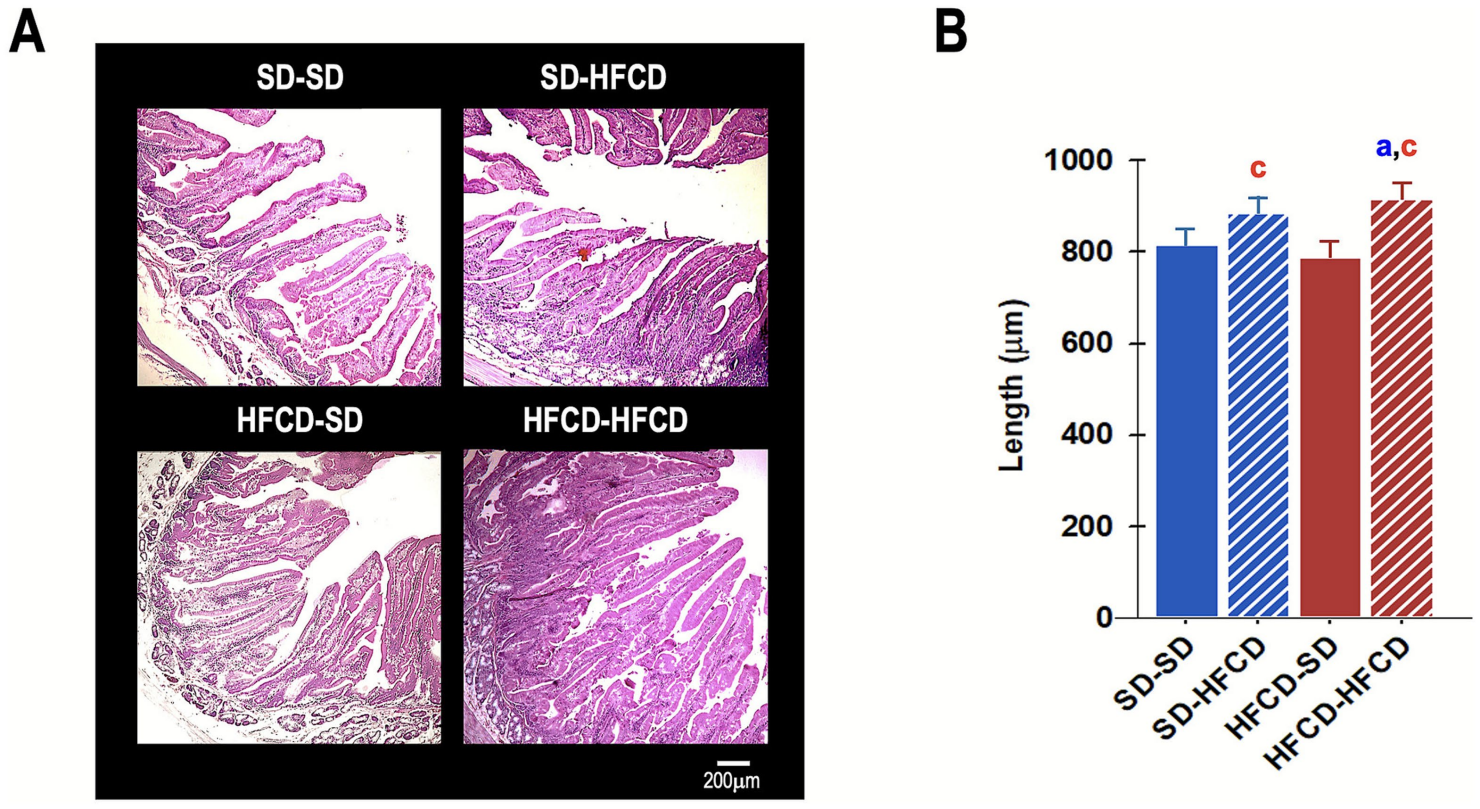
Histological evaluation of duodenal villi. **(A)** Photomicrograph of the duodenum sections and **(B)** quantitative analysis of villus length in the duodenum of adult rabbits obtained from mothers fed a standard diet (SD; blue bars) or a high-fat and high-carbohydrate diet (HFCD; red bars). In adulthood, the offspring were metabolically challenged with either the SD or the HFCD (striped blue and red bars). Experimental groups: SD/SD (*n* = 7), SD/HFCD (*n* = 7), HFCD/SD (*n* = 9), and HFCD/HFCD (*n* = 10). Sections were stained with hematoxylin–eosin (HE), magnification: 4×. ^a^ indicates *p* < 0.05 vs. SD-SD, ^c^ indicates *p* < 0.05 vs. HFCD-SD.

Regarding the structure and integrity of the intestinal epithelium, significant alterations were identified among the experimental groups. Significant changes in villus length [*F*_(3, 144)_ = 3.3; *p* = 0.02] were observed between the groups, and the HFCD–HFCD group exhibited a significant increase of 11.9% compared with the SD–SD group (Figure 6). Additionally, villus width also differed significantly across groups [*F*_(3, 133)_ = 5.0; *p* = 0.002]. *Post-hoc* comparisons indicated that, compared with the SD–HFCD and HFCD–HFCD groups, the HFCD–SD group showed a significant reduction in villus width (*p* = 0.03).

**Figure 7:**
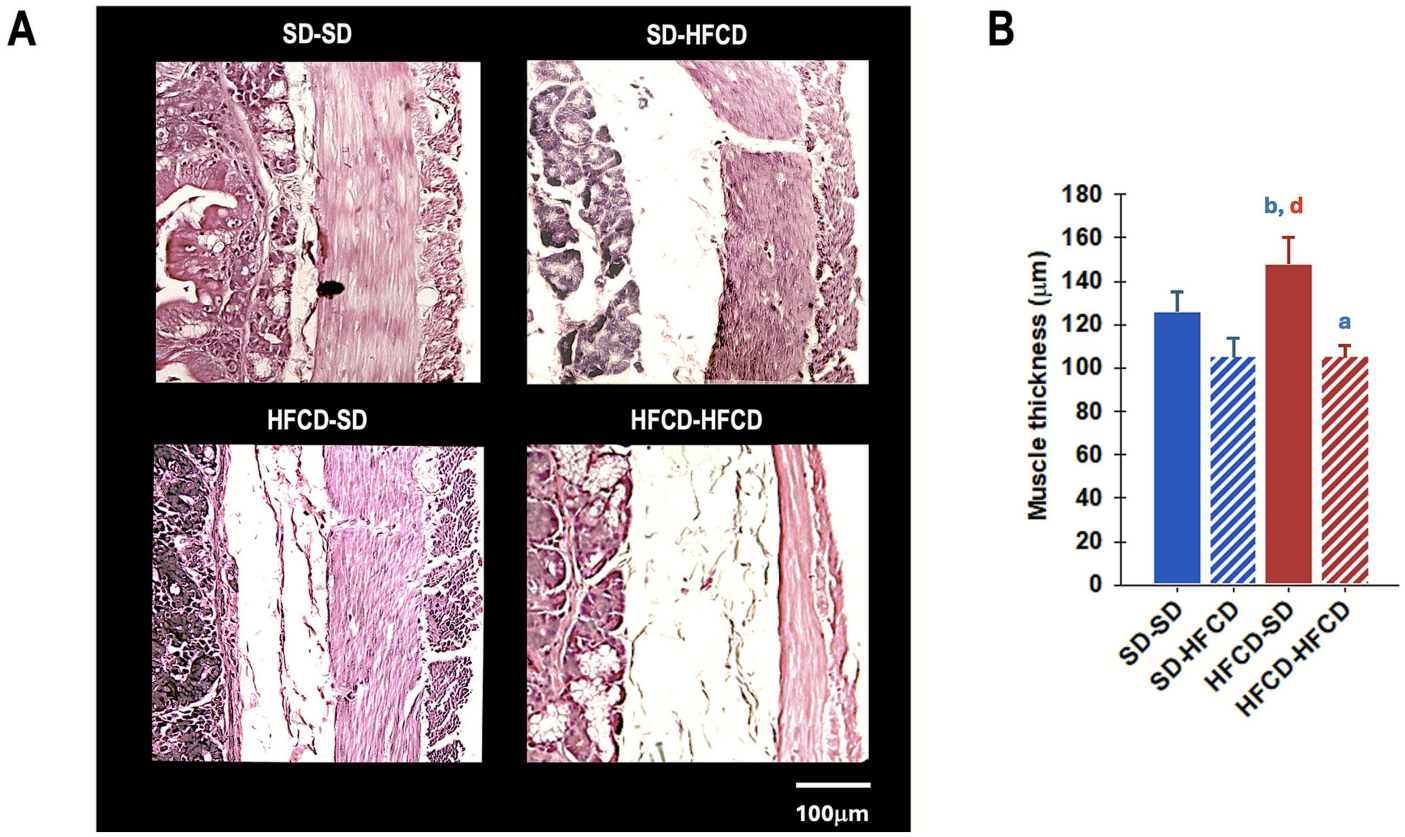
Duodenal muscularis propria thickness. **(A)** Representative photomicrograph of the duodenum sections and **(B)** quantification of the thickness of the muscle layer of the duodenum in rabbits obtained from mothers fed a standard diet (SD; blue bars) or a high-fat and high-carbohydrate diet (HFCD; red bars). In adulthood, the offspring were metabolically challenged with either the SD or the HFCD (striped blue and red bars). Experimental groups: SD/SD (*n* = 7), SD/HFCD (*n* = 7), HFCD/SD (*n* = 9), and HFCD/HFCD (*n* = 10). Sections were stained with hematoxylin–eosin (HE), magnification: 10×. ^a^ indicates *p* < 0.05 vs. SD-SD, ^b^ indicates *p* < 0.05 vs. SD-HFCD, ^d^ indicates *p* < 0.05 vs. HFCD-HFCD.

Additionally, significant differences in intestinal muscle thickness (Figure 7) were observed among the experimental groups [*F*_(3, 98)_ = 5.9; *p* < 0.001]. Compared with the SD–SD group, the HFCD–HFCD group exhibited an increase in muscle layer thickness of approximately 16.4%. Furthermore, the HFCD–SD group showed significantly reduced muscle thickness compared with both the SD–HFCD (*p* < 0.001) and HFCD–HFCD (*p* < 0.001) groups.

**Figure 8:**
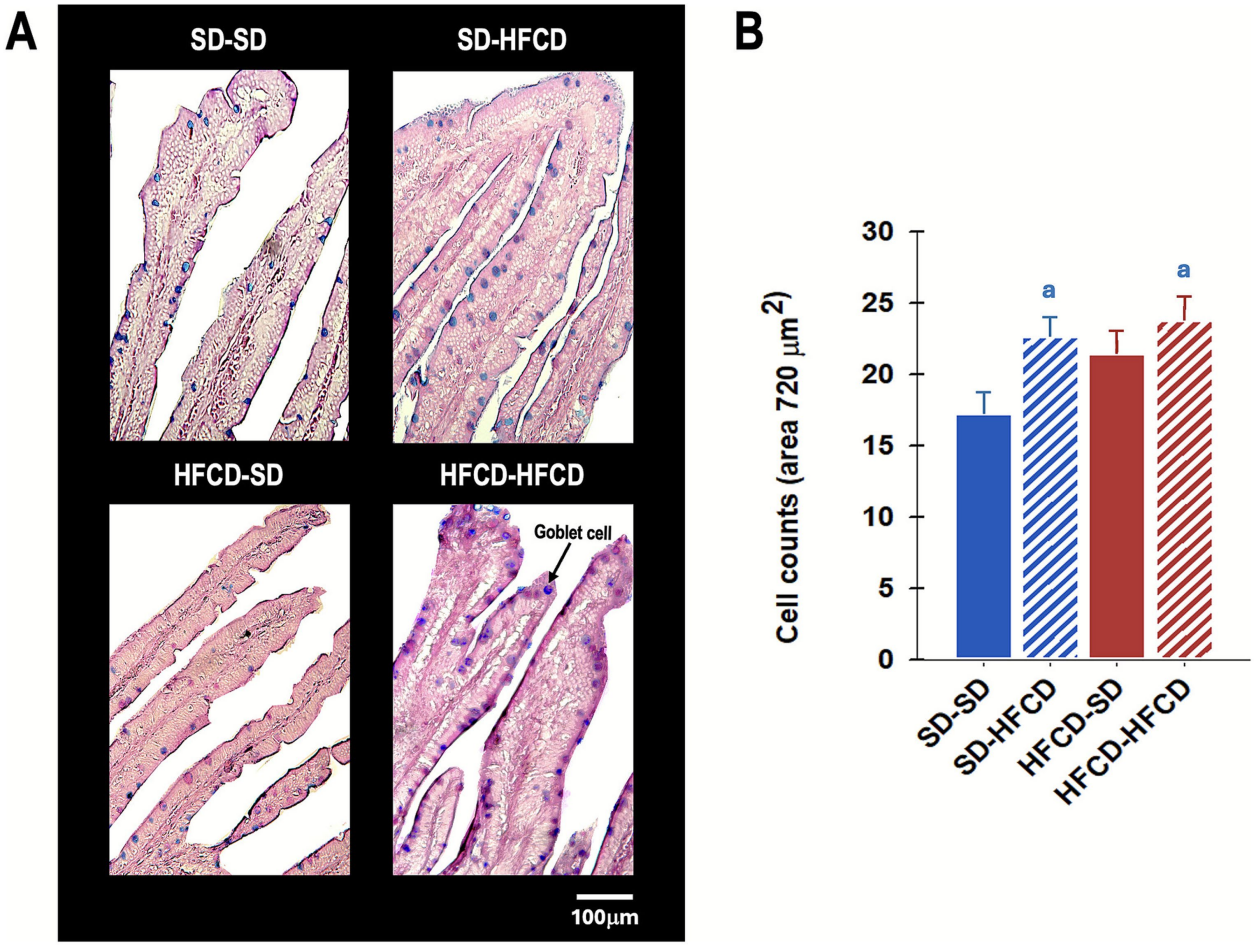
Goblet cell abundance in the duodenum. **(A)** Representative photomicrograph of the duodenum sections and **(B)** quantification of goblet cells of the duodenal epithelium in rabbits obtained from mothers fed a standard diet (SD; blue bars) or a high-fat and high-carbohydrate diet (HFCD; red bars). In adulthood, the offspring were metabolically challenged with either the SD or the HFCD (striped blue and red bars). Experimental groups: SD/SD (*n* = 7), SD/HFCD (*n* = 7), HFCD/SD (*n* = 9), and HFCD/HFCD (*n* = 10). Sections were stained with Periodic Acid–Schiff (PAS), magnification: 10 ×. ^a^ indicates *p* < 0.05 vs. SD-SD.

Regarding goblet cell quantification, significant differences were observed among the experimental groups [*F*_(3, 25)_ = 3.1; *p* < 0.04]. Compared with the SD–SD group, the SD–HFCD and HFCD–HFCD groups showed significantly greater numbers of goblet cells per villus, approximately 30.9 and 37.4%, respectively (Figure 8).

**Figure 9:**
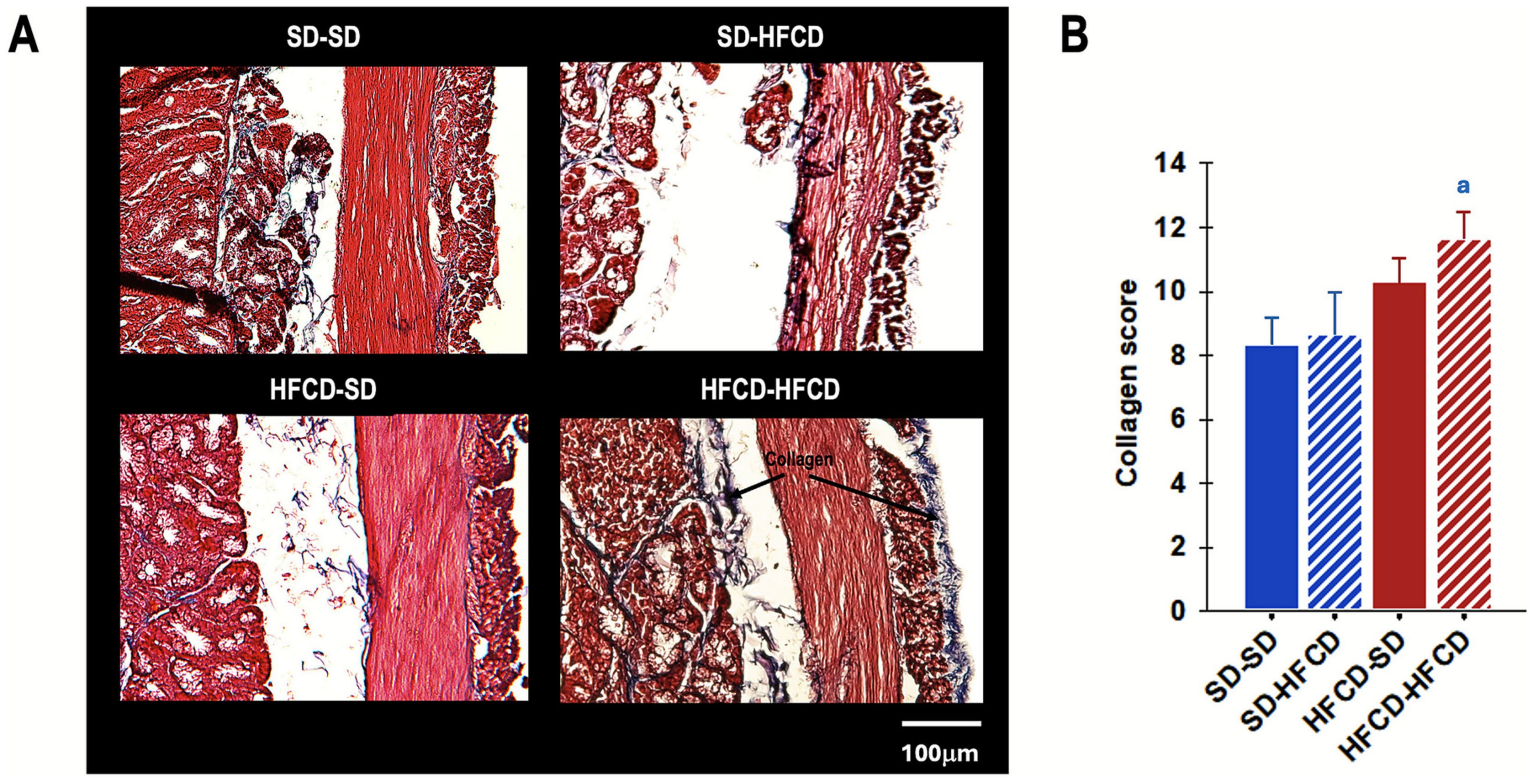
Collagen fiber accumulation in the duodenum. **(A)** Representative photomicrograph of collagen fibers in duodenum sections and **(B)** quantification of collagen deposition in the duodenum in rabbits obtained from mothers fed a standard diet (SD; blue bars) or a high-fat and high-carbohydrate diet (HFCD; red bars). In adulthood, the offspring were metabolically challenged with either the SD or the HFCD (striped blue and red bars). Experimental groups: SD/SD (*n* = 7), SD/HFCD (*n* = 7), HFCD/SD (*n* = 9), and HFCD/HFCD (*n* = 10). Sections were stained with Masson’s trichrome (MT), magnification: 10×. ^a^ indicates *p* < 0.05 vs. SD-SD.

In the quantification of Brunner’s gland area, significant differences were identified among the groups [*F*_(3, 739)_ = 2.7; *p* < 0.04], although no group showed significant differences relative to the control group (SD–SD). For the number of Brunner’s glands and crypt depth, no statistically significant differences were observed [*F*_(3, 31)_ = 1.2; *p* = 0.3; *F*_(3, 209)_ = 1.0; *p* = 0.4]. Finally, quantification of collagen fibers revealed significant group differences (*H* = 8.5; *p* = 0.04). The HFCD–HFCD group exhibited a significant increase in collagen fiber content, approximately 39.7% greater than that observed in the SD–SD group (Figure 9).

## 4 Discussion

Our findings demonstrate that chronic intake of a high-fat and high-carbohydrate diet (HFCD) during gestation, followed by a subsequent metabolic challenge with the same diet in adulthood, results in differential metabolic and histological effects in the liver and duodenum of rabbits. Histological alterations included villous elongation, thickening of the muscular layer, goblet cell proliferation, and increased collagen deposition in the intestine, accompanied by hepatic microvesicular changes and disruptions in energy metabolism. The effects of overnutrition have been extensively studied during pregnancy, lactation, and adulthood; few investigations have examined the combined impact of exposure during gestation and later in adult life—the period when metabolic disorders most commonly manifest. In this study, histological analysis of the liver and intestine following double exposure to an HFCD provides novel insight into dietary adaptability and the mechanisms underlying GLA function.

Rabbit models are recognized as reliable models for investigating the long-term effects of high-cholesterol diets, hyperlipidemia, and atherosclerosis development, due to their lipid metabolism, which closely resembles that of humans. Because rabbits exhibit high levels of apolipoprotein B and cholesterol ester transfer protein, a key regulator of reverse cholesterol transport (34), they are particularly susceptible to lipid-induced cardiovascular pathologies (35). In addition, the composition and distribution of adipose tissue in rabbits are comparable to those in humans, making them a valuable model for studying postprandial hypertriglyceridemia and visceral fat accumulation (36, 37). Consequently, the European rabbit is widely regarded as an excellent experimental model for investigating the mechanisms underlying metabolic syndrome and atherosclerosis (38).

Another relevant feature of this experimental model is its placental morphology. Rabbits possess a hemochorial placenta, in which the fetal chorion is in direct contact with maternal blood, allowing efficient exchange of gases, nutrients, and signaling molecules. This type of placenta is classified according to the number of tissue layers separating maternal and fetal blood. In humans, the placenta is hemomonochorial, characterized by a single trophoblastic layer. In contrast, rabbits have a hemodichorial placenta with two trophoblastic layers, whereas rodents, such as rats and mice, have a hemotrichorial placenta with three trophoblastic layers. The number of interposing layers between maternal and fetal tissues is a critical determinant of nutrient and metabolite transfer efficiency across the placental barrier (39). Beyond placental structure, rabbits offer additional advantages for studying reproductive physiology and fetal programming. They possess a larger number of blastomeres per blastocyst and yield higher embryo numbers, along with a well-characterized morphological and molecular profile of gastrulation stages, making them particularly suitable for developmental studies. The impact of maternal nutritional restriction on fetal development has been explored using rabbit models, as demonstrated by Lopez-Tello et al. (40), who investigated intrauterine growth restriction (IUGR) and found strong parallels with human responses.

Given these anatomical, physiological, and metabolic parallels, particularly in placental structure, intrauterine growth dynamics, and lipid metabolism, the European rabbit is a robust and reliable model for investigating nutritional alterations during gestation and their long-term effects on offspring.

Analysis of the metabolic profile revealed significant alterations in the group subjected to both prenatal exposure and postnatal metabolic challenge, as rabbits in the HFCD–HFCD group exhibited elevated serum levels of GLU and GLY. These findings are consistent with previous studies in rodent models, in which offspring of dams fed an HFD exhibited hyperglycemia and glucose intolerance upon re-exposure to a similar diet under conditions indicative of insulin resistance (41). The observed increase in GLU and GLY concentrations may reflect altered gluconeogenic activity, which is likely associated with impaired insulin signaling. Glycerol synthesis is primarily derived from glucose, and high-carbohydrate diets are known to upregulate the expression of glycerogenic enzymes (42, 43).

Alterations in lipid metabolism, particularly in CHOL and LDL-C levels, were evident in the SD–HFCD and HFCD–HFCD groups, suggesting that dietary imbalance during adulthood has a significant effect, promoting hypercholesterolemia and elevated LDL-C concentrations. In contrast, rodents from dams exposed to an HFD during gestation and lactation show elevated serum lipid levels, including TG and CHOL, as early as 3 weeks of age (44). The discrepancies between our findings and those in rodent models may be attributed to differences in diet composition, duration and timing of exposure (lactation versus adulthood), and species-specific metabolic responses, particularly the heightened sensitivity of rabbits to lipid metabolism disturbances (45). Notably, TG levels appeared resistant to dietary modulation, which is consistent with previous observations of minimal changes in TG concentrations despite prolonged HFCD exposure in rabbits (29).

Additionally, analysis of liver damage biomarkers revealed unexpectedly lower levels of AST and ALT in the HFCD–HFCD group. These results contrast with typical findings in models of Western-style diet intake, where elevated transaminase levels are commonly associated with hepatic injury. Interestingly, clinical studies in patients with chronic kidney disease have reported that reduced serum transaminase levels are correlated with a decreased glomerular filtration rate (46). These findings raise the possibility that long-term HFCD exposure may contribute to renal dysfunction in this group, and further analysis is needed to corroborate these findings.

Regarding correlation analyses, a negative correlation between AST and LDL levels was observed in subjects with acute myocardial infarction, as reported by Reddy et al. (47). Low levels of AST have been associated with various conditions, including vitamin B6 deficiency in older adults and individuals with chronic alcohol consumption, as well as liver and kidney diseases and certain inflammatory conditions (48). Therefore, low AST levels may reflect increased liver or renal risk associated with vitamin B6 deficiency. On the other hand, AST showed a positive association with TG levels, and a positive correlation has been observed between AST and metabolic syndrome parameters, such as TG, body mass index, waist circumference, and systolic/diastolic blood pressure, showing a potential link between metabolic disturbances and high levels of AST (49). Both high and low AST levels are indicators of cardio-metabolic risk. Elevated levels of lipids (cholesterol and LDL) in combination with high GGT levels have been suggested as indicators of the severity of lipid-induced hepatic dysfunction and associated complications (50).

Furthermore, analysis of liver damage markers revealed significant increases in plasma TB and LPS levels in the HFCD–SD group. In previous studies, elevated TB levels have been associated with liver immaturity in offspring exposed to a high-carbohydrate diet during the uterine stage. Bilirubin has been shown to bind directly to peroxisome proliferator-activated receptor alpha (PPARα). Furthermore, analysis of liver damage markers revealed significant increases in plasma TB and LPS levels in the HFCD–SD group. Previous studies have associated elevated TB levels with hepatic immaturity in offspring exposed to a high-carbohydrate diet during the intrauterine stage. Bilirubin has been shown to bind directly to peroxisome proliferator-activated receptor alpha (PPARα), thereby enhancing the transcription of genes involved in lipid oxidation, such as Cpt1, Ucp1, and Adrb3, and promoting mitochondrial biogenesis (51). The interaction between bilirubin and PPARα reduces lipid storage by upregulating the *β*-oxidation pathway through increased expression of FGF21 (fibroblast growth factor 21), CPT1 (carnitine palmitoyltransferase I), pAMPK (phosphorylated AMP-activated protein kinase), and pAkt (phosphorylated Akt). In contrast, obesity or consumption of a high-fat diet leads to decreased levels of bilirubin and PPARα, resulting in elevated FAS (fatty acid synthase) and ACC (acetyl-CoA carboxylase) expression, which promotes triglyceride accumulation (52). Additionally, bilirubin has been suggested to exert a protective effect against metabolic-associated fatty liver disease (MAFLD), potentially mediated through improvements in insulin sensitivity and reductions in systemic chronic inflammation (53).

The observed increases in bilirubin and LPS levels in the group exposed to gestational overnutrition may be associated with metabolic programming events, potentially mediated by alterations in the gut microbiota (54). In rabbits, probiotic supplementation has been shown to reduce serum bilirubin levels, suggesting a modulatory role of the microbiota in hepatic function (55).

Although an increase in intestinal permeability was anticipated in the groups exposed to a postnatal dietary challenge, these groups did not show increased serum LPS levels, a marker of gut barrier dysfunction. In animal models, increased circulating LPS levels have been implicated in the disruption of gut permeability. Moreover, LPS modulates lipid metabolism; for example, it can influence systemic lipid homeostasis, as LPS injection in mice reduces plasma levels of HDL-C (56).

We observed a significant increase in circulating LPS levels in the HFCD–SD group, but not in the HFCD–HFCD group. In line with the DOHaD hypothesis, this difference may be attributed to a mismatch between the intrauterine nutritional environment and dietary conditions during adulthood (57). Interestingly, LPS may exert context-dependent metabolic benefits in the HFCD–SD group, as previous evidence suggests that, under certain conditions, LPS can ameliorate inflammation and metabolic disturbances by activating the TLR4–IL-23–IL-22 pathway (58). Collectively, these findings underscore the pivotal role of maternal nutrition in shaping offspring development and the resulting differential metabolic responses to dietary challenges later in life. Future studies should aim to elucidate the molecular mechanisms underlying the potential beneficial effects of LPS on metabolism.

In this study, both the HFCD–HFCD and SD–HFCD groups developed microvesicular steatosis, a pathological hallmark associated with hepatocellular hypertrophy, hepatic dysfunction, and progression to non-alcoholic fatty liver disease (NAFLD, now MAFLD) (59). It is possible that microvesicular steatosis could be an indicator of NAFLD severity in obese individuals. In support of this, Tandra and collaborators reported a significant association between microvesicle accumulation and the severity of NAFLD in liver biopsies, including increased fibrosis localized to Zone 3 of the hepatic acinus and to the periportal and portal regions (60). Interestingly, in our model, fibrosis was not observed in Zone 3, which may be attributed to the limited duration (30 days) of the postnatal dietary challenge; prolonged exposure may be required to initiate fibrogenesis in the rabbit liver. This interpretation is consistent with previous studies that failed to detect fibrotic changes in rabbits, even after more than 6 months of dietary intervention (61). Nevertheless, previous animal models have consistently shown that prenatal exposure to a high-fat diet predisposes offspring to the development of NAFLD (10, 62, 63).

With respect to intestinal histology, rabbits in the HFCD–HFCD group exhibited notable histomorphological alterations, including increased villus height, increased goblet cell density, and evidence of fibrosis, accompanied by reduced muscularis thickness. Previous reports have demonstrated that diets high in simple carbohydrates (e.g., fructose) and saturated fat can induce elongation of the small intestine, possibly as an adaptive response to increase nutrient absorption and facilitate lipid accumulation (64). Evidence from animal models has identified potential molecular mechanisms underlying alterations induced by high-fat and high-carbohydrate diets, including increased phosphorylation of glycogen synthase kinase 3 beta (GSK-3*β*) and activation of the Wnt/β-catenin signaling pathway. These processes lead to enhanced proliferation of intestinal epithelial cells, such as elongation of the villi, which results in greater absorptive capacity (65, 66). Both the GSK-3β and Wnt/β-catenin pathways are sensitive to dietary modulation and play pivotal roles in tumorigenesis. They are upregulated under conditions such as colon cancer, contributing to increased cell proliferation and histological remodeling that expand the intestinal absorptive surface area, thereby promoting metabolic alterations and weight gain (67).

The morphological changes observed in the HFCD–HFCD rabbits, such as elongation of the intestinal villi, may represent early structural adaptations that potentiate long-term metabolic dysregulation, including an increased risk of obesity and associated comorbidities.

In addition, there was a significant increase in the number of goblet cells in the gut of the groups exposed to the nutritional challenge during adulthood. Goblet cells play crucial roles in the synthesis and secretion of mucins, which constitute the protective mucus layer of the intestinal epithelium. Previous studies have demonstrated that short-term exposure to an HFD induces goblet cell hyperplasia, a response that is associated with increased susceptibility to pathogenic infections and intestinal inflammation in murine models (68).

Our results showed that exposure to HFCD prenatally and during a metabolic challenge induced a significant increase in duodenal collagen deposition. Longer exposure to an HFD has been previously associated with structural alterations in the intestinal barrier, characterized by inflammatory cell infiltration and enhanced collagen accumulation in the colonic wall, thereby contributing to intestinal dysfunction (69). In addition, maternal obesity promotes inflammation and fibrosis in the large intestine of ovine fetuses and offspring, supporting the idea that gestational programming affects intestinal tissue development (70).

Finally, the group exposed to HFCD prenatally and subjected to a metabolic challenge in adulthood showed a significant reduction in intestinal muscle thickness. Although evidence regarding the association between reductions in intestinal muscularis propria thickness and nutritional status is limited, studies using the experimental autoimmune encephalomyelitis (EAE) model have demonstrated increased proinflammatory Th1/Th17 cytokine expression and reduced regulatory T-cell populations in the gut lamina propria, Peyer’s patches, and mesenteric lymph nodes, accompanied by a decrease in duodenal muscle thickness (71). This phenotype has been associated with inflammation and intestinal fibrosis, particularly in disorders such as inflammatory bowel disease, highlighting the critical role of gestational immune programming in intestinal development (72, 73).

Our findings suggest that muscle atrophy may be associated with HFCD intake. Intestinal smooth muscle, similar to other muscle types, relies on actin–myosin filament interactions for contraction. Muscle atrophy involves a reduction in muscle cell size, primarily due to the accelerated degradation of contractile proteins such as actin and myosin (74). In our study, the HFCD included increased amounts of carbohydrates and fats, along with a reduction in protein content (29). Under conditions such as malnutrition or chronic disease, cellular signaling pathways that promote muscle protein degradation are activated. Catabolic stimuli induce the expression of muscle-specific E3 ubiquitin ligases, including MuRF1 and Atrogin-1/MAFbx, which target selective substrates for ubiquitination and subsequent degradation by the 26S proteasome, leading to atrophy across muscle types, including intestinal smooth muscle (75). Thus, diet-induced disruption of the anabolic–catabolic balance could promote excessive protein degradation and subsequent smooth muscle atrophy. Moreover, HFCD intake has been associated with intestinal barrier alterations through the downregulation of tight junction proteins (occludin, ZO-1, and claudin-4) and gut dysbiosis, which exacerbate gastrointestinal symptoms in colitis models (76). However, a direct causal association between atrophy and barrier dysfunction has not yet been demonstrated. The impact of overnutrition on intestinal barrier function appears to depend strongly on the timing of exposure, underscoring the importance of temporally sensitive periods in gastrointestinal and systemic development. Future studies should aim to clarify the influence of maternal and adult overnutrition on GLA, with particular attention to long-term hepatic and intestinal pathophysiology. The impact of overnutrition on intestinal barrier function appears to be highly dependent on the timing of exposure, reinforcing the relevance of temporally sensitive periods in gastrointestinal and systemic development. Future studies should aim to clarify the influence of maternal and adult overnutrition on GLA, with particular attention to long-term hepatic and intestinal pathophysiology.

In conclusion, our findings demonstrate that exposure to a high-fat and high-carbohydrate diet during gestation and/or adulthood induces significant metabolic disturbances and histopathological alterations in both the liver and the intestinal epithelium. These effects appear to depend on the timing and duration of exposure and may have long-term implications for offspring health. Our results underscore the importance of investigating HFCD-induced metabolic and histological changes across critical developmental windows to advance our understanding of GLA-mediated metabolic regulation.

## Data Availability

The raw data supporting the conclusions of this article will be made available by the authors, without undue reservation.

## Glossary

ALT: alanine aminotransferase
ANOVA: analysis of variance
AST: aspartate aminotransferase
CB: conjugated bilirubin
CHOL: total cholesterol
DOHaD: Developmental Origins of Health and Disease
EAE: experimental autoimmune encephalomyelitis
ELISA: enzyme-linked immunosorbent assay
FDR: false discovery rate
FFA: free fatty acids
GGT: gamma-glutamyl transferase
GLA: gut–liver axis
GLU: glucose
GLY: glycerol
HDL-C: high-density lipoprotein cholesterol
HE: hematoxylin–eosin
HFD: high-fat diets
HFCD: high-fat and high-carbohydrate diet
IBD: inflammatory bowel disease
LDL-C: low-density lipoprotein cholesterol
LPS: lipopolysaccharides
LSD: least significant difference
MAFLD: metabolic-associated fatty liver disease
MT: Masson’s trichrome
NAFLD: non-alcoholic fatty liver disease
NIH: National Institutes of Health
ORO: Oil Red O
PPARα: peroxisome proliferator-activated receptor alpha
PAS: Periodic Acid–Schiff
PND: postnatal day
SD: standard diet
TB: total bilirubin
TG: triglycerides
